# Identification and Management of Testicular Mesothelioma Identified Intraoperatively: A Case Series

**DOI:** 10.7759/cureus.61190

**Published:** 2024-05-27

**Authors:** Tara Sweeney, Isabelle Doan, Robert L Grubb

**Affiliations:** 1 Department of Urology, Medical University of South Carolina, Charleston, USA

**Keywords:** pelvic lymph node dissection, complex hydrocele, orchiectomy, testicular mesothelioma, testicular mass

## Abstract

Testicular mesothelioma lacks the characteristic presentation of testicular malignancy and often has normal biomarkers at the time of diagnosis causing this malignancy to be overlooked and diagnosed intraoperatively during elective scrotal surgery. We present two cases of testicular mesothelioma that were diagnosed incidentally during hydrocelectomy. These cases emphasize the importance of considering testicular mesothelioma during hydrocele and scrotal mass workup and demonstrate the need for standardized guidelines for the management of testicular mesothelioma.

## Introduction

Mesothelioma is a neoplasm that arises from the simple squamous epithelium that lines serous cavities such as the pleura and the tunica vaginalis [1]. Mesotheliomas can be grouped into three pathological subtypes: well-differentiated papillary mesothelioma, mesothelioma of uncertain malignant potential, and malignant mesothelioma [2,3]. Although pleural mesothelioma accounts for most mesotheliomas, mesothelioma of the tunica vaginalis testis can rarely occur, with fewer than 1% of malignant mesotheliomas being testicular in origin [4,5].

Testicular mesothelioma lacks the characteristic presentation of testicular malignancy and often has normal biomarkers at the time of diagnosis causing this malignancy to be overlooked and diagnosed intraoperatively during elective scrotal surgery [6,7]. According to the National Comprehensive Cancer Network (NCCN) guidelines, the first step of diagnosis and treatment of testicular malignancy should be with a radical orchiectomy through an inguinal incision to prevent tumor ablation and allow for a confirmatory diagnosis [8]. Given the rarity and nonspecific preoperative presentation of testicular mesothelioma, the diagnosis is often not considered until the tunica vaginalis has been violated. This convolutes postoperative management as there are few cases in the literature and no standardized guidelines on the management of testicular mesothelioma.

We present two cases of testicular mesothelioma that were diagnosed incidentally during hydrocelectomy. These cases emphasize the importance of considering testicular mesothelioma during hydrocele and scrotal mass workup and demonstrate the need for standardized guidelines for the management of testicular mesothelioma.

## Case presentation

Patient 1

**Figure 1:**
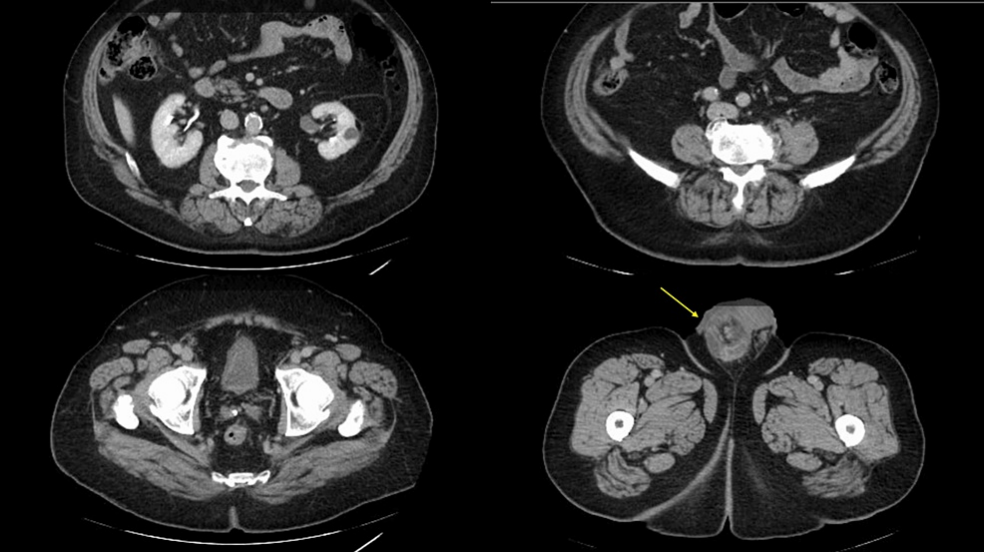
CT of the abdomen and pelvis showing right inguinal hernia without metastatic disease (arrow) CT: computed tomography

A 77-year-old male with no remarkable urological history or asbestos exposure initially presented to an outside urology office with right-sided testicular swelling and was diagnosed with a large right hydrocele and small left hydrocele on scrotal ultrasound. He underwent a right hydrocelectomy, in which he was noted to have nodules throughout the right tunica vaginalis. A biopsy was taken of the right testicle, and hydrocelectomy was completed at the time of the biopsy. On final pathology, he was found to have an atypical mesothelial proliferation. However, malignant versus papillary mesothelioma could not be determined. He was therefore referred to our institution, in which staging imaging was completed. He had a computed tomography (CT) of the abdomen and pelvis with contrast only notable for a right inguinal hernia and a CT of the chest negative for cardiopulmonary disease (Figure 1).

Given concerns for malignant mesothelioma, he underwent a right radical inguinal orchiectomy and hemiscrotectomy. The final pathology showed epithelioid-type malignant mesothelioma involving the paratesticular soft tissue, rete testis, and spermatic cord with a positive margin at the spermatic cord. The immunohistochemical stain was positive for BAP1 expression in the tumor cells.

He subsequently underwent bilateral retroperitoneal lymph node dissection with spermatic cord excision. He was found to have metastatic disease with several positive lymph nodes and a positive proximal spermatic cord margin.

The patient was referred to medical oncology for assistance in managing metastatic disease. It was determined that he would not be able to tolerate cisplatin and was recommended to undergo adjuvant carboplatin/pemetrexed. It was also discussed that he would be considered for a combination of platinum-based chemotherapy and checkpoint inhibitors, such as nivolumab, if interval CT scans were to show overt metastatic disease.

He completed adjuvant chemotherapy treatment with carboplatin/pemetrexed with interval CT of the chest, abdomen, and pelvic absent for discrete mass or overt metastatic disease. He will continue interval imaging every six months to assess for disease progression and has not had progression of disease one year after diagnosis.

Patient 2

**Figure 2:**
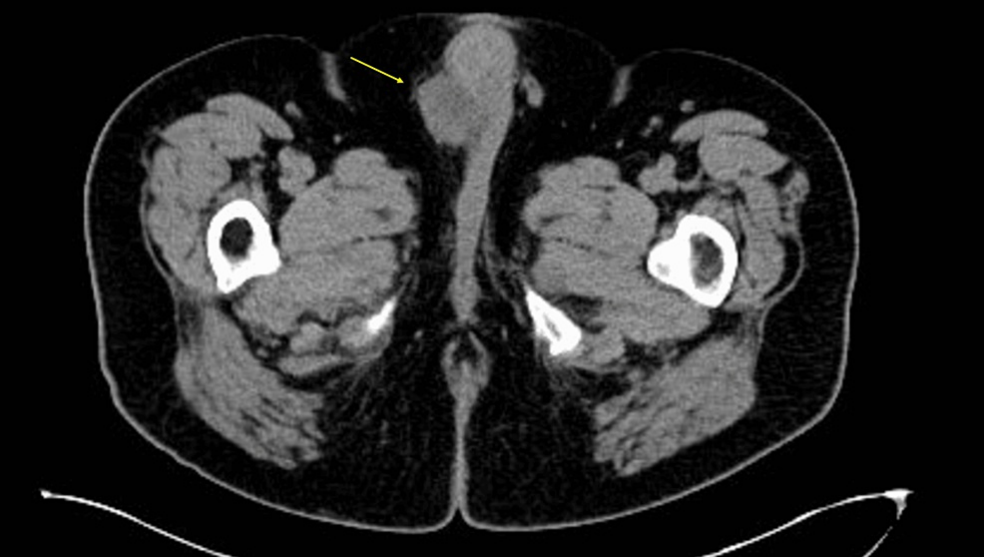
CT of the abdomen and pelvis demonstrating incidentally found partially visualized scrotal lesion (arrow) CT: computed tomography

A 75-year-old male with a history of high-risk non-muscle-invasive bladder cancer and no asbestos exposure was found to have right testicular swelling during a clinic visit for bladder cancer surveillance. He underwent scrotal ultrasound, which showed a 1 cm right spermatocele and an 8 cm right hydrocele as well as a 3 cm left hydrocele. He had previously had the right-sided hydrocele drained by a urologist at a different institution and reported that the hydrocele returned within a month. He had abdominal cross-sectional imaging done as part of his bladder cancer surveillance imaging, which only partially showed the right hydrocele (Figure 2).

He underwent hydrocelectomy through a transscrotal approach, in which the testicular lining was noted to have multiple small nodules with a cobblestone appearance. Frozen sections were sent intraoperatively, which were concerning for a neoplastic process. Management options were discussed with the patient's wife intraoperatively, in which the decision was made to move forward with an inguinal right radical orchiectomy with spermatic cord excision. Final pathology showed malignant mesothelioma without disease in the spermatic cord. He underwent chest imaging, which did not show cardiopulmonary involvement.

The patient was planned to undergo bilateral retroperitoneal lymph node dissection but has since moved out of state and plans to complete surgery at an outside institution.

## Discussion

Malignant mesothelioma of the tunica vaginalis testis lacks specific symptoms, making a preoperative diagnosis challenging [9]. Although initial presentation is often a recurrent or chronic hydrocele, it has not been determined whether long-term hydrocele is a risk factor for mesothelioma. Therefore, recurrent hydroceles do not necessarily warrant higher suspicion for testicular mesothelioma [10]. Asbestos exposure may be associated with testicular mesothelioma, but this association is not as strong as it is known to be for pleural mesotheliomas, and reviews of extrapleural mesotheliomas note that there is insufficient evidence to link tunica vaginalis testis mesothelioma to asbestos exposure [5,11]. This is further supported by our case series in which neither patient had an obvious risk factor for developing testicular mesothelioma such as a history of asbestos exposure.

In a population-based study looking at the incidence of patients with testicular mesothelioma in the United States from 1973 to 2015, there were only noted to be 113 patients diagnosed with testicular mesothelioma. The five-year disease-specific survival of these patients was 58%, with a 10-year disease-specific survival of 45% [12]. In a systematic review of patients with testicular mesothelioma, it was found that the disease-specific survival for non-metastatic malignant mesothelioma of the tunica vaginalis was 23 months versus 18 months if the disease was metastatic [13].

A scrotal ultrasound is often the imaging modality that is used for the workup of a painless scrotal mass or concern for hydrocele on a physical examination. Ultrasound of mesotheliomas may demonstrate exophytic and papillary tumors as well as heterogeneous or hypoechogenic fluid; however, the imaging modality is overall limited by the pathological characteristics of mesothelioma. Mesothelioma lesions may be small, solitary, and multiple, which is not readily visualized on ultrasound [7]. The limitation of ultrasound in visualizing these exophytic neoplasms is clearly demonstrated in our case series, in which both patients underwent ultrasound with findings most consistent with a benign hydrocele.

Long-term survival potential is dependent on the initial radical orchiectomy through an inguinal approach to remove the tumor and surrounding tissue, with hemiscrotectomy if there is a concern for skin involvement [14]. Similar to other malignant mesotheliomas, testicular mesothelioma runs an aggressive course with possible metastasis to the lung, abdominal organs, and retroperitoneal and inguinal lymph nodes [9]. Intraoperatively, multiple nodules lining the hydrocele sac should raise suspicion of tunica vaginalis mesothelioma with further concern for possible infiltration of the spermatic cord. The prognosis and disease course become convoluted when a testicular neoplasm is discovered intraoperatively and during an elective scrotal surgery, which often uses a transscrotal approach, increasing the potential for tumor metastasis and positive margins. In a literature review of testicular mesothelioma, a 36% local recurrence rate of testicular mesothelioma after simple hydrocelectomy for malignant mesothelioma was found compared to an 11% local recurrence rate when managed with radical inguinal orchiectomy. Similarly, in subsequent reviews, the median survival rate was about 23 months, with the rate decreasing to 14 months with recurrence [7].

In both described patients, scrotal exploration was performed through a scrotal incision, putting the patient at risk for tumor dissemination through testicular lymphatics and delaying diagnosis of spermatic cord involvement [15]. It is unknown if the extensive metastatic disease found in our first patient was due to dissemination during the drainage of the hydrocele and subsequent hydrocelectomy. It can also be suspected that the extent of the patient's metastatic disease could have been more effectively managed if it was known that there was involvement of his spermatic cord earlier in his disease course. The risk of dissemination in our second patient from the initial transscrotal incision is also not known. Although the need for further intervention and an inguinal incision was noted and completed intraoperatively, there are no existing guidelines on decreasing the risk of dissemination after tunica vaginalis violation in a situation of an incidentally found mesothelioma.

When suspecting testicular mesothelioma during a transscrotal exploration, the current practice is to perform an inguinal orchiectomy if able to obtain intraoperative consent than to perform a second surgery with a hemiscrotectomy to obtain negative margins [7]. Although there are no existing recommendations on subsequent retroperitoneal lymph node dissection for an intraoperatively diagnosed testicular mesothelioma, the presence of primary metastasis is reported in 15% of cases with worse survival outcomes for patients with metastatic testicular mesothelioma [7,13]. Therefore, retroperitoneal lymph node dissection provides the patient with the most efficient course of diagnosis and management if metastatic disease is found.

## Conclusions

Our two cases demonstrate the importance of primary surgical treatment when testicular mesothelioma is suspected and the lack of guidelines present when testicular mesothelioma is identified intraoperatively. In accordance with findings from our case series and existing literature, we can state that performing inguinal orchiectomy as a second surgery or intraoperatively if able to get appropriate consent can ensure that a confirmatory diagnosis can be made. We would also state that when there is a concern for potential tumor dissemination, retroperitoneal lymph node dissection following diagnosis is advantageous to the patient as this allows for more prompt systemic therapy and can decrease morbidity and mortality than if metastatic disease is not found or diagnosed later in the disease course.
